# Sarcoid‐resembling granulomatous lung disease secondary to occupational magnetite iron dust exposure

**DOI:** 10.1002/rcr2.331

**Published:** 2018-05-17

**Authors:** Lewis Xiao, Anil Kookana, Robert McClure, Subash Heraganahally

**Affiliations:** ^1^ Respiratory Medicine Royal Darwin Hospital Darwin Australia; ^2^ Medical Imaging Royal Darwin Hospital Darwin Australia; ^3^ Western Diagnostic Pathology Perth Australia

**Keywords:** Granuloma, iron ore, magnetite, occupational, sarcoidosis

## Abstract

Non‐caseating granulomatous pulmonary conditions resembling sarcoidosis secondary to industrial/occupation exposure to magnetite iron ore dusts have been rarely documented in the literature. This is a case report of a 58‐year‐old blast crew member involved in iron ore/magnetite mining who presented with a 12‐month history of chronic dry cough. High‐resolution computed tomography revealed bilateral interstitial opacities. Lung biopsy demonstrated sarcoid‐like granulomatous inflammation. Oral corticosteroid treatment improved the cough. Radiological features did not resolve despite treatment and yet remained stable following no subsequent exposure to iron mining dust.

## Introduction

Sarcoidosis is one of many well‐recognized causes of granulomatous lung disease encountered in clinical practice. However, it is important to recognize that there are several other both infectious and non‐infectious conditions that can also give rise to granulomatous lung disease, and accurate diagnosis of non‐sarcoid granulomatous lung disease can be challenging 1. Occupational beryllium exposure has been a well‐recognized ethology in the pathogenesis of granulomatous lung disease.

Although there are literature reports suggesting that exposure to industrial dusts can also give rise to granulomatous lung disease, granulomatous pulmonary conditions resembling sarcoidosis secondary to exposure to iron ore dusts have been rarely documented 2. In this case report, we describe a patient who was diagnosed with granulomatous lung disease resembling sarcoid following exposure to magnetite, an iron ore industrial dust.

## Case Report

**Figure 1:**
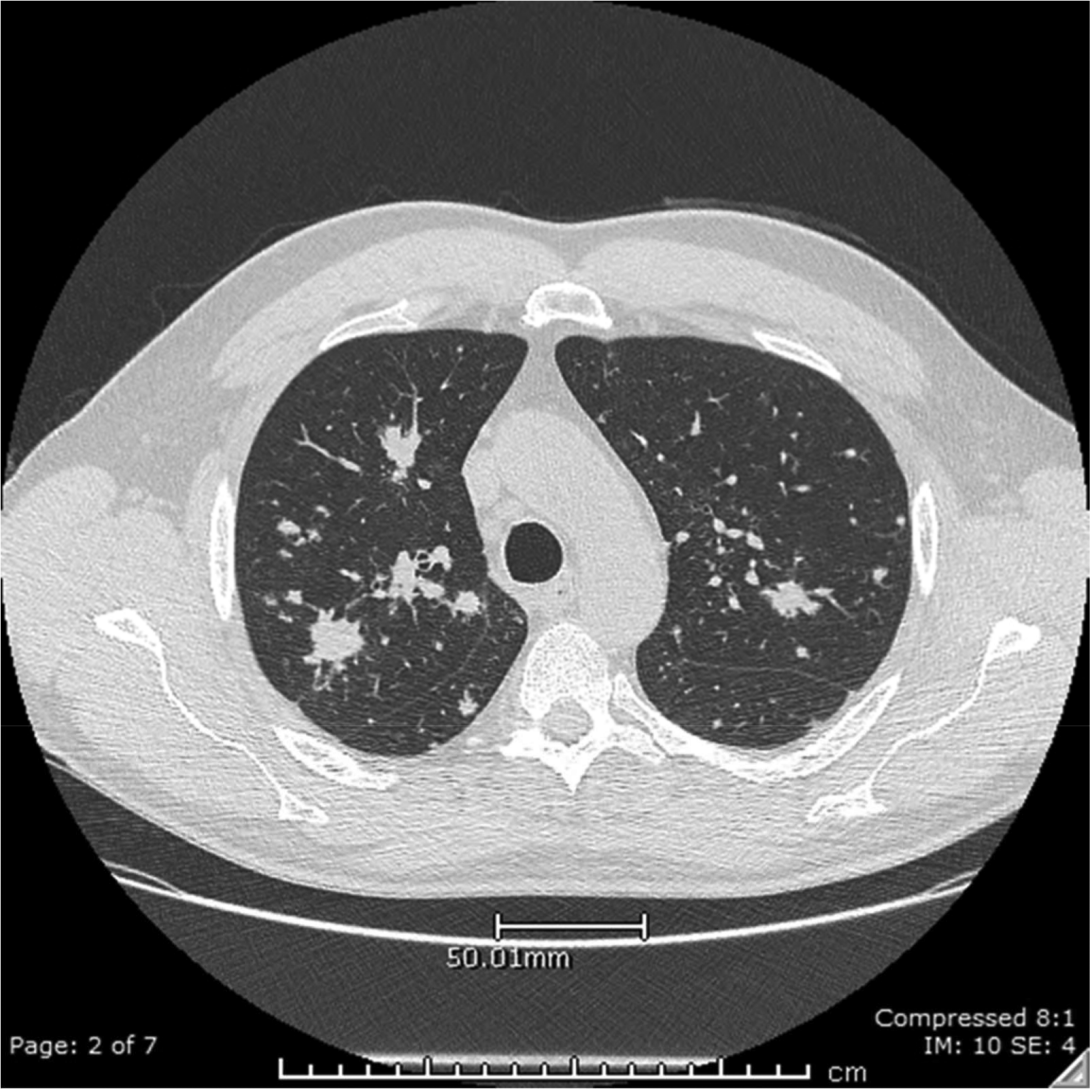
High resolution computed tomography demonstrating bilateral interstitial opacities.

A 58‐year‐old man presented with a 12‐month history of dry cough but was otherwise asymptomatic with no constitutional symptoms. His past medical history only included reflux disease for which he was on a regular proton pump inhibitor. He was a non‐smoker and denied illicit drug taking. There was no recent travel history or contact with tuberculosis nor exposure to asbestos. He had a three‐year occupational history within the iron ore/magnetite mining industry as a blast crew member and therefore had been directly exposed to iron/magnetite dust. He used the respiratory anti‐dust mask only intermittently during his work. General physical and respiratory system examination was unremarkable. High‐resolution chest computed tomography (HRCT) (Fig. 1) demonstrated bilateral interstitial opacities throughout both lungs, most pronounced in the apical segments of the upper and lower lobes with relative sparing of the basal and peripheral lung fiends. There was a mild mediastinum and hilar adenopathy measuring 6 mm in size. Nodal or parenchymal calcification was not apparent.

**Figure 2:**
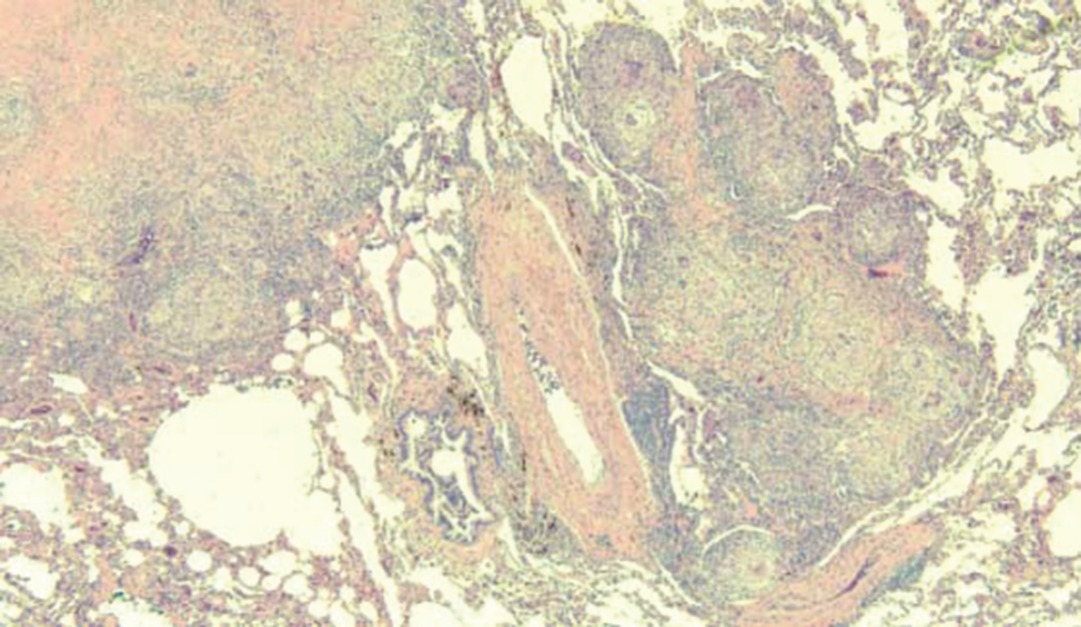
Histology showing non‐necrotizing epithelioid granulomas.

A panel of basic blood tests, including haemoglobin, electrolytes, and liver and renal function tests, were unremarkable. More extensive investigations, including HIV, hepatitis screens, rheumatoid factor, ENA, ANA, angiotensin‐converting enzyme (ACE), and 24‐h urinary calcium excretion, were in the normal range. Pulmonary function showed a forced vital capacity (FVC) of 88% (3.79 L), FEV1 of 72% (2.36 L), and FEV1/FVC ratio of 82% (0.622 L), with no acute bronchodilator response. Gas transfer (DLCO) was normal. To rule out other possible inflammatory/infective causes, a bronchoscopy with washings and bronchoalveolar lavage (BAL) was performed, which showed bronchial epithelial cells, macrophages, and neutrophils; no malignant cells were recognized. Approximately 1% of the cells identified on BAL were of lymphocytes by CD 45: SS gating, and a majority of cells appeared to be T cells with a CD4:CD 8 ratio of 3:1. Acid‐fast bacilli were not identified, and both fungus and mycobacterial cultures were negative. Transbronchial lung biopsy was inconclusive; hence, he underwent a right upper lobe biopsy using a video‐assisted thorascopy procedure. The biopsy demonstrated scattered, non‐necrotizing epithelioid granulomas of varying sizes, measuring up to 6 mm, comprising epithelioid cells, langhans cells, and occasional lymphocytes (T cells). The lesions were noted be nodular and were mainly located in the lung interstitium adjacent to the small airway, blood vessels, connective tissue septa, or immediately beneath the pleura. The lesions also demonstrated central hyalinised sclerosis. No definitive Schumann bodies or asteroid bodies were identified. Within the nodules, deposits of anthrocotic pigments were seen. No other foreign body material was identified, including silica. No atypical mycobacterium, fungal elements, or other microorganisms were identified with appropriate stating (Fig. 2). Mineral particles in the biopsy specimen were not examined due to a lack of such diagnostic facilities at our centre.

As a trial of treatment, the patient was initiated on oral corticosteroids, which helped slightly with the cough; however, on subsequent imaging, the bilateral opacities remained stable, demonstrating neither resolution nor progression. Lung function showed no significant change. The patient has had no further exposure to industrial dust for almost one year from his initial presentation.

## Discussion

Sarcoidosis is a multisystem disease, primarily affecting the lung in 90% of cases, with the development of non‐caseating granulomas seen as the trademark histopathological feature. The exact aetiology of sarcoidosis is still unknown, and therefore, it is essentially a diagnosis of exclusion. Certain investigations can promote the diagnosis of sarcoidosis over other granulomatous diseases, namely, increased serum ACE, an absence of identifiable pathogens, hypercalcaemia, and Schaumann bodies positively identified on biopsy tissue, which were all negative in our case. We strongly believe that the granulomatous reaction in this case is triggered by exposure to occupational magnetite iron dust rather than conventional sarcoidosis.

It has been documented that the inhalation of industrial dusts, including magnetite, can lead to a wide variety of lung pathologies such as diffuse interstitial fibrosis and pneumoconiosis and including sarcoid‐like granulomatous lesions and foreign body reactions, similar to our patient 2. It has been hypothesized that exposure to occupational or environmental metal dust acts as a trigger for sarcoid‐like granulomatous reactions, as noted in metal workers 2, 3. One other report recounted a patient presenting with granulomatous pulmonary nodules with a seven‐year history of occupational brushing and polishing of surgical materials 4, and interestingly, “sarcoid‐like” granulomatous pulmonary disease has been identified in firefighters involved with the recovery missions of the 11 September 2001 World Trade Centre collapse in New York 5. Researchers identified a significant increase in the incidence of pulmonary parenchymal abnormalities, mediastinal adenopathy, and sarcoid‐like granulomas in firefighters directly involved in rescue at Ground Zero compared to those who were not.

Occupational dust‐related non‐sarcoidosis granulomatous lung disease has been reported previously, although, admittedly, these studies are rare, especially following iron ore mineral exposure. Such patients are a diagnostic dilemma. We believe this case report will prompt further research into occupational non‐sarcoid granulatomous lung diseases.

### Disclosure Statement

Appropriate written informed consent was obtained for publication of this case report and accompanying images.
